# Supplementary material for: Lobar emphysema ratio of more than 1% in the lobe with lung cancer as poor predictor for recurrence and overall survival in patients with stage I non-small cell lung cancer
Source: PLoS One. 2023 Feb 14;18(2):e0281715. doi: 10.1371/journal.pone.0281715 (PMC9928128; doi:10.1371/journal.pone.0281715)
Supplement: Attachment — Submitted filename: response to reviewers.docx [file pone.0281715.s002.docx]

When submitting your revision, we need you to address these additional requirements.

1. Please ensure that your manuscript meets PLOS ONE's style requirements, including those for file naming.

Title Page (Page 1, Line 9, 12)

I added the information about affiliation “Gyeongsang National University Hospital”

Title Page (Page 1, Line 14)

I changed the word ‘pichola’ to ‘MJP’.

2. In your Data Availability statement, you have not specified where the minimal data set underlying the results described in your manuscript can be found. PLOS defines a study's minimal data set as the underlying data used to reach the conclusions drawn in the manuscript and any additional data required to replicate the reported study findings in their entirety. All PLOS journals require that the minimal data set be made fully available. For more information about our data policy, please see <http://journals.plos.org/plosone/s/data-availability.>

; We uploaded our minimal data set as supporting information file.

We will update your Data Availability statement to reflect the information you provide in your cover letter.

3. Please upload a new copy of Figure 1b as the detail is not clear. Please follow the link for more information:

; Figure 1b is a multipanel image. So I changed figure 1b into figure 1b,c,d for better understanding. And I added some information in the figure legend about figure 1b,c,d.

I added the word “in axial and coronal images” in Line 131, Page 6.

I added the following sentence in Line 134-135, Page 6

“The lung densities in whole lung and each pulmonary lobe were presented using the density histogram analysis. The lobar emphysema ratio in the left lower lobe was 16.591, and he was confirmed to have adenocarcinoma after lobectomy.”

4. Please review your reference list to ensure that it is complete and correct. If you have cited papers that have been retracted, please include the rationale for doing so in the manuscript text, or remove these references and replace them with relevant current references. Any changes to the reference list should be mentioned in the rebuttal letter that accompanies your revised manuscript. If you need to cite a retracted article, indicate the article’s retracted status in the References list and also include a citation and full reference for the retraction notice.

Reviewer #1: Very well written. Very few studies have been published focusing on the correlation between lobar emphysema and stage I non-small cell lung cancer. There are a few studies on a similar topic, and this study is consistent with published literature.

; Thank you for your nice comments.

Reviewer #2: Well done study. Conclusion was expected as patients with more severe emphysema will have poor outcome in general. Given the higher rates of active smoker and worse Fev1 in the HER group, they could potentially be a confounding factor when looking at DFS and OS. Since we are discussing emphysema in this study it would have been better if diffusion capacity (DLCO) was also reported for each category to look for another potential confounding factor.

; I agree with your opinion. I reviewed some related articles, added the following paragraph in the discussion section. (Line 260-271 Page 14)

“Although airflow obstruction has been known as an independent risk factor for lung cancer [23,24], its prognostic significance is still controversial in patients with early-stage lung cancer. Lopez et al. reported that airflow obstruction is a poor prognostic factor for 2-year survival in stage I lung cancer [28]. However, Ueda et al. reported that airflow obstruction is not a significant poor prognostic factor for 5-year OS and DFS in patients with lung cancer, mostly early-stage lung cancer [13], these findings are consistent with our results. Further research are required to verify the prognostic value of airflow obstruction in patients with lung cancer.

Smoking status is a well-known poor prognostic factor for survival in patients with lung cancer [29,30], furthermore smoking cessation after diagnosis lowers the mortality in patients with early-stage lung cancer [31]. In contrast, our results indicated that smoking status is not a significant prognostic factor for OS and DFS. Compared to previous large-scale investigations, the number of study population and mortality rate were low in our study. These factors might affect our study's results.

”

I also mentioned the diffusion capacity in the limitation section. (line 277-279, Page 14, 15)

“Fourth, although we did not gather information on diffusion capacity in all patients, it may be a complicating factor for survival in patients with lung cancer. Patients with normal PFT in our institution are optional to undergo the diffusion capacity test.”

Reviewer #3: Very nicely done study.
Manuscript is well written and easy to follow.

I had one comment

1) Did the authors look at degree of airflow obstruction(FEV1%) as a predictor of decreased DFS or OS? It will add to the findings of references 21,23,24 in the text.

;

Thank you for your comments. Previous studies indicated that the prognostic role of airflow obstruction (FEV1%) is controversial in patients with lung cancer.

The prognostic impact of airflow obstruction is uncertain in reference 21. The reference of 23, 24 revealed the association between airflow obstruction and risk of lung cancer.

I reviewed the other related articles and added this paragraph in the discussion section (Line 260-266, Page 14).

“Although airflow obstruction has been known as an independent risk factor for lung cancer [23,24], its prognostic significance is still controversial in patients with early-stage lung cancer. Lopez et al. reported that airflow obstruction is a poor prognostic factor for 2-year survival in stage I lung cancer [28]. However, Ueda et al. reported that airflow obstruction is not a significant poor prognostic factor for 5-year OS and DFS in patients with lung cancer, mostly early-stage lung cancer [13], these findings are consistent with our results. Further research are required to verify the prognostic value of airflow obstruction in patients with lung cancer.”

---------------------------

Finally I corrected some minor error.

1. I changed the word from “nonesmall” to “non-small” in the reference 21, Line 339

Bishawi M, Moore W, Bilfinger T. Severity of emphysema predicts location of lung cancer and 5-y survival of patients with stage I nonesmall cell lung cancer. J Surg Res. 2013;184: 1–5. doi:10.1016/j.jss.2013.05.081

🡪

Bishawi M, Moore W, Bilfinger T. Severity of emphysema predicts location of lung cancer and 5-y survival of patients with stage I non-small cell lung cancer. J Surg Res. 2013;184: 1–5. doi:10.1016/j.jss.2013.05.081

2. I changed the word “emphysema quantification” to “density histogram analysis” in Line 226, Page 12.
